# Commentary: Retrospective and prospective perspectives on zoonotic brucellosis

**DOI:** 10.3389/fmicb.2019.01859

**Published:** 2019-08-14

**Authors:** Cathy Kneipp, Richard Malik, Siobhan M. Mor, Anke K. Wiethoelter

**Affiliations:** ^1^Faculty of Veterinary and Agricultural Sciences, University of Melbourne, Melbourne, VIC, Australia; ^2^Centre for Veterinary Education, University of Sydney, Sydney, NSW, Australia; ^3^School of Animal and Veterinary Sciences, Charles Sturt University, Wagga Wagga, NSW, Australia; ^4^Institute of Infection and Global Health, University of Liverpool, Liverpool, United Kingdom

**Keywords:** *Brucella*, Brucellosis, *Brucella canis*, Australia, dog, fox, *Brucella suis*

We are writing in response to the article by Moreno (2014) titled “Retrospective and prospective perspectives on zoonotic brucellosis”, which contains two important errors regarding the status of *Brucella canis* in dogs and wildlife reservoirs of *Brucella* strains in Australia.

Moreno (2014) states “Canine brucellosis has just been recently found in domestic dogs in Australia” (page 7) citing Gardner and Reichel (1997) and Hofer et al. (2012). The article by Gardner and Reichel (1997) actually documents the absence of *B. canis* in the neighboring country of New Zealand, while Hofer et al. (2012) reported the first domestic detection of *B. canis* in a breeding kennel in Austria (Europe), not Australia. In fact, *B. canis* has never been reported in Australia or New Zealand (DAF, 2018a). We note that Australia has recently experienced a surge of cases of brucellosis in dogs, but these were caused by *B. suis*, not *B. canis* (Mor et al., 2016; James et al., 2017).

Moreno (2014) also states that “*Brucella* strains have been isolated in rodents and foxes in Australia” (page 7) citing Tiller et al. (2010) and Al Dahouk et al. (2012). No foxes from Australia were tested in these studies and there is no literature published to support detection of *Brucella* spp. in foxes in Australia. The article cited by Moreno (2014) to support evidence in foxes is Al Dahouk et al. (2012) which refers to another study by Scholz et al. (2009). The latter reports the isolation of *B. microti* from mandibular lymph nodes of red foxes (*Vulpes vulpes*) in Lower Austria (again, not Australia).

Strains of *Brucella* have been isolated from rodents in Australia. Tiller et al. (2010) re-examined *Brucella* strains collected by Cook et al. (1966) in North Queensland from three species of rodents, *Rattus assimilis, Melomys cervinipes*, and *M. lutillus*. These strains were originally reported as *B. suis* biovar 3 by Cook et al. (1966) but Tiller et al. (2010) “strongly” suggested they were a new atypical *Brucella* species. Wattam et al. (2012) subsequently found similarities between the atypical Australian rodent strains and two novel strains (BO1^T^ and BO2) from atypical human infections.

Australia was declared free of *B. abortus* in 1989 after a long and successful eradication program and currently only two of the 10 well-characterized species of *Brucella* occur naturally in Australia. Specifically, *B. ovis* (non-zoonotic) is found only in sheep where it causes epididymal lesions and reproductive losses. *Brucella suis* biovar 1 is also endemic in feral pigs in certain regions and can be transmitted from feral pigs to humans and dogs (DAF, 2018a). The latter species is largely absent from the domestic swine herd in Australia (Rogers et al., 1989; DAF, 2018b).

To maintain Australia's status and international reputation of being free from other forms of brucellosis it is important to correct inaccurate statements such as those made by Moreno (2014).

## Author Contributions

CK identified the error and wrote the commentary with input from SM, RM, and AW. All authors critically reviewed and approved the final version.

### Conflict of Interest Statement

The authors declare that the research was conducted in the absence of any commercial or financial relationships that could be construed as a potential conflict of interest.
